# *Mycobacterium tuberculosis* LipE Has a Lipase/Esterase Activity and Is Important for Intracellular Growth and *In Vivo* Infection

**DOI:** 10.1128/IAI.00750-19

**Published:** 2019-12-17

**Authors:** Dong Yang, Shaoji Li, Jennifer Stabenow, Lillian Zalduondo, Ying Kong

**Affiliations:** a Department of Microbiology, Immunology, and Biochemistry, University of Tennessee Health Science Center, Memphis, Tennessee, USA; b Regional Biocontainment Laboratory, University of Tennessee Health Science Center, Memphis, Tennessee, USA; Weill Cornell Medical College

**Keywords:** *Mycobacterium tuberculosis*, lipase/esterases, LipE, intracellular survival, macrophage

## Abstract

Mycobacterium tuberculosis Rv3775 (LipE) was annotated as a putative lipase. However, its lipase activity has never been characterized, and its precise role in tuberculosis (TB) pathogenesis has not been thoroughly studied to date. We overexpressed and purified the recombinant LipE (rLipE) protein and demonstrated that LipE has a lipase/esterase activity. rLipE prefers medium-chain ester substrates, with the maximal activity on hexanoate. Its activity is the highest at 40°C and pH 9. We determined that rLipE hydrolyzes trioctanoate. Using site-directed mutagenesis, we confirmed that the predicted putative activity triad residues Ser97, Gly342, and His363 are essential for the lipase activity of rLipE. The expression of the *lipE* gene was induced under stressed conditions mimicking M. tuberculosis’ intracellular niche. The gene-disrupting mutation of *lipE* led to significantly reduced bacterial growth inside THP-1 cells and human peripheral blood mononuclear cell-derived macrophages and attenuated M. tuberculosis infection in mice (with ∼8-fold bacterial load reduction in mouse lungs). Our data suggest that LipE functions as a lipase and is important for M. tuberculosis intracellular growth and *in vivo* infection.

## Abstract (precis)

Mycobacterium tuberculosis Rv3775 (LipE) was annotated as a putative lipase. However, its lipase activity has never been characterized, and its precise role in tuberculosis (TB) pathogenesis has not been thoroughly studied to date. We overexpressed and purified the recombinant LipE (rLipE) protein and demonstrated that LipE has a lipase/esterase activity. rLipE prefers medium-chain ester substrates, with the maximal activity on hexanoate. Its activity is the highest at 40°C and pH 9.

## INTRODUCTION

Emerging literature has discovered that Mycobacterium tuberculosis utilizes lipids and fatty acids (FAs) as important nutrients during infection. After phagocytosis by the alveolar macrophages, M. tuberculosis can manipulate macrophage to accumulate lipid bodies and form a foamy phenotype (1). During infection, M. tuberculosis relies on its lipases to hydrolyze host lipids to release FAs by catalyzing the hydrolysis of ester bonds in long-chain acylglycerols (2, 3). Genomic sequencing of M. tuberculosis H37Rv and CDC1551 strains predicted that M. tuberculosis possesses more than 250 genes related to lipid metabolism (4, 5). Among them, 24 lipid/ester hydrolases of M. tuberculosis were annotated belonging to the “Lip” family (LipC to LipZ) (4, 5). Some of the Lip family lipase/esterase activities have been characterized (6–16). Six additional hypothetical genes of M. tuberculosis encoding esterases have also been identified, and they contain the pentapeptide motif “GxSxG” shared by most of the Lip family proteins (17). Some proteins involved in lipid metabolism of M. tuberculosis are also virulence related, and mutations of them lead to attenuated phenotypes in cell and animal infection. These include mycolic acid synthases (18), trehalose synthases (19), polyketide synthases (20), FA-coenzyme A (CoA) synthases (21), isocitrate lyases (22), phospholipases (23), acyl-CoA dehydrogenases (24), lipid carriers (24), and lipid transporters (25–28). Some lipases also play critical roles in M. tuberculosis virulence. For example, the gene-disrupting mutation of *lipF* caused bacterial load reduction in lungs of mice (29, 30). Mutation of another lipase/esterase, Rv2224c, also caused decreased bacterial load in mice (10). Overexpression of LipY in M. bovis bacillus Calmette-Guérin impaired immune protection against infection in mice (14). Because lipid/ester catabolism is an important requirement for M. tuberculosis infection and persistence in hosts, functional characterization of the specific lipases/esterases in M. tuberculosis lipid/ester catabolism pathways provides an opportunity to discover new mechanisms of tuberculosis (TB) pathogenesis.

Dutta et al. used a pool of 326 M. tuberculosis mutants to infect a nonhuman primate model and identified mutations in 108 M. tuberculosis genes that were attenuated for *in vivo* growth (31). LipE was listed as one of them. However, the precise role of LipE in TB pathogenesis has not been thoroughly studied to date. Although the lipase activities of a few Lip family lipases have been characterized, the activity and function of LipE in M. tuberculosis lipid catabolism remain unexplored. In this study, we characterized the lipase/esterase activity of recombinant LipE (rLipE) and evaluated its catalytic triad and its hydrolysis of triglycerides. We also evaluated its transcriptional expression under stressed conditions that mimic the M. tuberculosis intracellular niche in phagosome. Finally, we defined the impact of LipE on M. tuberculosis intracellular growth in macrophages and on M. tuberculosis *in vivo* infection.

## RESULTS

### Amino acid sequence analysis of Rv3775 (LipE) and homology 3D model of LipE.

**FIG 1:**
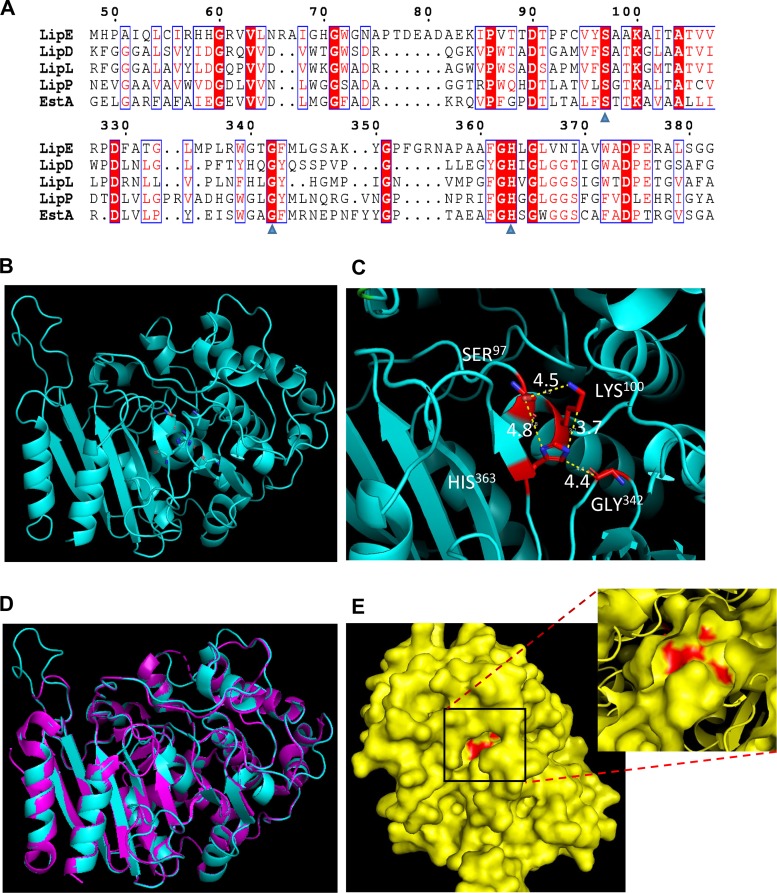
Protein sequence analysis of LipE and predicted 3D structure model of LipE. (A) Protein sequence alignment of LipE with LipD, LipL, and LipP and esterase A (PDB 5GKV.1) from C. crescentus. The putative active triad amino acids of LipE are indicated by blue triangles. (B) Predicted LipE 3D structure model. (C) Magnification of the putative active triad area in the 3D structure model. Distances between atoms on the putative active triad amino acids are shown as yellow dashes (in Å). (D) Alignment of LipE structure with the structure of C. crescentus esterase A (PDB 5GKV.1). Cyan, LipE; pink, 5GKV.1. (E) LipE 3D structure surface view. The predicted active triad is indicated in red.

We obtained the amino acid sequence of Rv3775 (415 amino acids [aa], 45.3 kDa) from Tuberculist, in which Rv3775 is annotated as LipE and predicted to belong to the Lip family lipases. We constructed a phylogenetic tree of the 24 M. tuberculosis Lip family proteins. This tree showed that LipE might be evolutionarily close to LipD, LipL, and LipP (see Fig. S1 in the supplemental material). We then aligned the amino acid sequence of LipE with the sequences of LipD, LipL, and LipP and EstA of Caulobacter crescentus. EstA from C. crescentus was the template for constructing a homology three-dimensional (3D) model of LipE as described below. The sequence alignment revealed that LipE has an SxxK motif at aa 97 to 100, which is conserved in LipD, LipL, LipP, and EstA of C. crescentus (Fig. 1A). Homologous known crystal structures that displayed the maximum query coverage and sequence identity with LipE were chosen as the templates for generating the 3D model structures in a SWISS-MODEL workspace. Three model structures were generated based on homology to other bacterial lipases. They were further evaluated and ranked by measuring the distance between the catalytic residues and structural alignment with the templates using PyMOL. The 3D model that had the lowest root mean square deviation (RMSD) value and hydrogen bond distance of the catalytic residues between the predicted model and the template was selected as the final model. The results showed that the EstA (PDB 5GKV.1) from C. crescentus had the highest similarity with the LipE sequence (41 identical amino acids, ranging from aa 26 to 405), and the 3D structure model of LipE using the EstA as a template had the lowest RMSD value (RMSD = 0.144). This predicted 3D structure of LipE is composed of seven β-strands, ten α-helices, and six 3_10_ helices (Fig. 1B). Combining the protein sequence alignment result with the 3D structure model, we predicted Ser^97^, Gly^342^, and His^363^ residues as the putative active triad for LipE (Fig. 1C).

### Biochemical characterization of LipE and determination of its catalytic triad.

**FIG 2:**
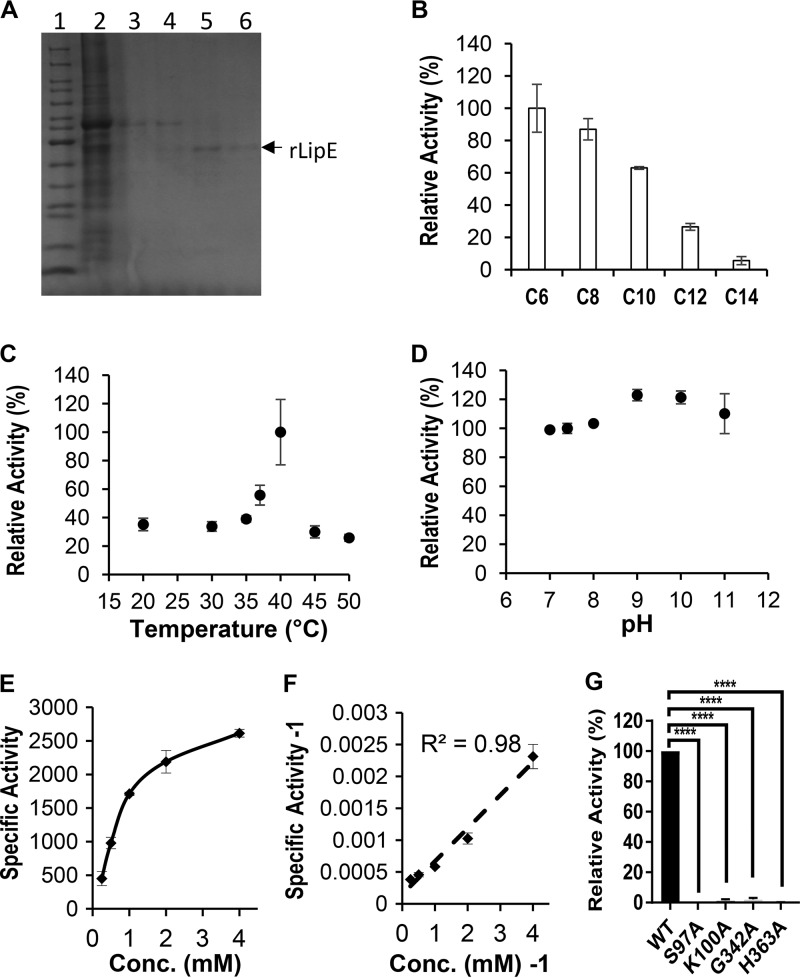
Purification of rLipE and analysis of LipE lipase/esterase activity. (A) SDS-PAGE analysis of rLipE eluted by various concentrations of imidazole from the His tag Ni column. Lanes: 1, protein molecular weight marker (PageRulerM); 2, protein extracts run through the Ni column with 40 mM imidazole buffer; 3 to 6, samples eluted from the Ni column with 62.5, 125, 250, and 500 mM imidazole, respectively. (B) Lipase relative activity of rLipE toward *p*-NP esters with various chain lengths (C6, caproate; C8, caprylate; C10, caprate; C12, laurate; and C14, myristate). The activity with *p*-NP-C6 was set as 100%. (C) Effect of temperature on lipase activity of rLipE using *p*-NP-C6 as a substrate at pH 7.4. The activity at 40°C was set as 100%. (D) Effect of pH on lipase activity of rLipE using *p*-NP-C6 as a substrate at 37°C. The activity at pH 7.4 was set as 100%. (E) Specific activity (U/mg) of rLipE over a series of *p*-NP-C6 concentrations. (F) Lineweaver-Burk (double-reciprocal) plot of specific activity^−1^ against the *p*-NP-C6 concentration^−1^. The values represent means ± the standard deviations (SD) of three independent experiments. (G) Verification of the active triad by site-directed mutagenesis. The wild-type or site-directed mutant rLipE was incubated with *p*-NP-C6 at 37°C and pH 7.4. The activity of the wild-type rLipE was set as 100%. ****, *P* < 0.0001 (one-way analysis of variance [ANOVA], followed by Dunnett’s multiple-comparison test). The values represent means ± the SD of three independent experiments.

We overexpressed LipE as a His-tagged fusion protein in M. smegmatis. The samples were analyzed by SDS-PAGE, and the results showed that the purified rLipE has a molecular mass of ∼45 kDa as predicted based on its amino acid sequence (Fig. 2A). An enzymatic assay of rLipE was performed using various *p*-nitrophenyl ester (*p*-NP-ester) substrates, with the *p*-NP-esters in the absence of rLipE as negative controls. We found that rLipE preferentially hydrolyzed medium-chain *p*-NP-esters: *p*-NP-C6, -C8, and -C10 had low activities with long-chain *p*-NP-C12 and -C14 and had no activities with *p*-NP-C2, -C4, -C16, and -C18 (Fig. 2B). Its optimal temperature was 40°C (Fig. 2C). rLipE had high activity in a wide range of pH values from pH 7 to 11, with the optimal pH 9, but no activity at pH 6 or lower (Fig. 2D). We determined its dynamic parameters with the *p*-NP-C6 as follows: specific activity, 560.95 ± 16.61 U/mg; *K_m_*, 711.58 ± 54.63 μM; *V*_max_, 6,756.76 ± 717.49 mM/min (using Lineweaver-Burk plot) (Fig. 2E and F). Our phylogenetic analysis indicated that LipE is evolutionarily close to LipD and LipL. The active triad residues in LipD were reported as Ser^102^, Asp^342^, and His^369^ (13), whereas in LipL the key catalytic amino acid residues were identified as Gly^50^, Ser^88^, and Lys^91^ (32). Based on our protein sequence alignment result and the 3D model of LipE, we predicted Ser^97^, Gly^342^, and His^363^ residues as the putative active triad for LipE (Fig. 1C and E). We conducted site-directed mutagenesis to generate single amino acid substitution mutations for the predicted active site residues Ser^97^, Gly^342^, and His^363^ to Ala and extracted the mutant rLipE proteins (Fig. S2). Because Ser^97^, Lys^100^, and His^363^ are predicted as catalytic residues of LipE for its β-lactamase activity (shown below), we also constructed a K100A mutant rLipE protein to investigate its impact on the lipase activity of LipE. G342A and K100A mutations led to enzymatic activities of LipE reduced by >95%, and S97A and H363A mutations completely demolished the lipase activity of LipE, indicating that Ser^97^, Lys^100^, Gly^342^, and His^363^ are essential for LipE lipase activity (Fig. 2G).

### LipE is capable of hydrolyzing triglycerides.

**FIG 3:**
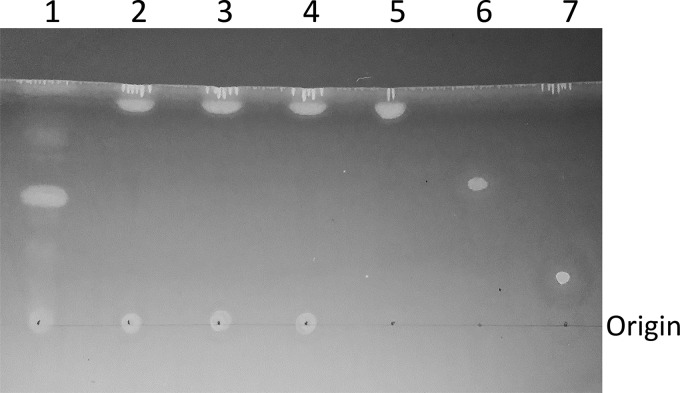
rLipE is capable of hydrolyzing trioctanoate. TLC analysis of hydrolysis of trioctanoate by rLipE was performed. Lanes: 1, trioctanoate + wild-type rLipE; 2, trioctanoate + S97A rLipE; 3, trioctanoate + G342A rLipE; 4, trioctanoate + H363A rLipE; 5, trioctanoate standard; 6, dioctanoate standard; 7, monooctanoate standard. Incubation time, 30 min.

We determined the activity of LipE on triglyceride hydrolysis using thin-layer chromatography (TLC) with trioctanoate as a substrate. Monooctanoate and dioctanoate were used as standards in this assay. The TLC results revealed that rLipE can hydrolyze trioctanoate into dioctanoate (Fig. 3), implying that LipE possesses triacylglycerol acyl hydrolase activity. The three mutant rLipEs (S97A, G342A, and H363A) cannot hydrolyze trioctanoate, indicating that these three residues are essential for the activity of triacylglycerol hydrolysis.

### LipE hydrolyzes fluorocillin green, a β-lactamase substrate.

**FIG 4:**
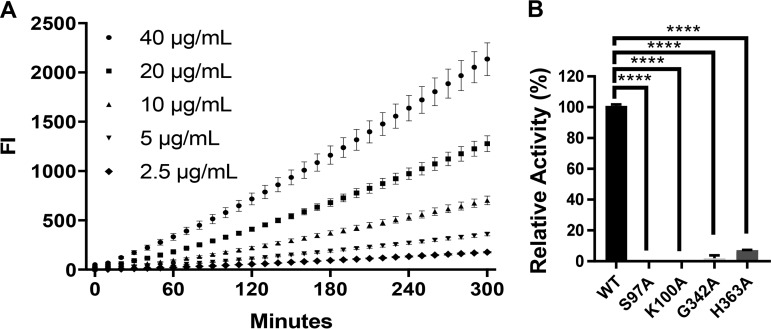
rLipE hydrolyzes fluorocillin green, a β-lactamase substrate. (A) Kinetics of rLipE on fluorocillin green. Five concentrations (2.5 to 40 μg/ml) of fluorocillin green were incubated with rLipE (10 μg) at 37°C. The kinetics of rLipE on fluorocillin green were measured at an excitation wavelength of 485 nm and an emission wavelength of 528 nm. (B) Determination of LipE active residues for its β-lactamase activity by site-directed mutagenesis. Wild-type or mutant rLipE (10 μg) was incubated with 10 μg/ml fluorocillin green at 37°C for 2 h. ****, *P* < 0.0001 (one-way ANOVA, followed by Dunnett’s multiple-comparison test). The values represent means ± the SD of three independent experiments.

We investigated the homology of the LipE amino acid sequence using Protein-BLAST and found that it has homologies to class A β-lactamase-related serine hydrolase. We determined its β-lactamase activity using a fluorogenic β-lactamase substrate, fluorocillin green, which has two attached cephalosporin moieties. The fluorocillin green sample without rLipE served as a negative control to normalize the signal of samples with rLipE. The results demonstrated that rLipE can hydrolyze fluorocillin green with a dose-response effect, indicating that it can cleave the cephalosporin ring structure in fluorocillin green (Fig. 4A). We determined its *k*_cat_ on fluorocillin green as 7.7685 ± 0.4527 s^−1^. We then analyzed its amino acid sequence and identified three SxxK motifs (97SAAK100, 131SHGK134, and 385SSGK388) and one HLG motif (363HLG365) conserved in β-lactamases . Combining this information with the 3D model, we predicted the catalytic residues of LipE for its β-lactamase activity as Ser^97^, Lys^100^, and His^363^. We verified this hypothesis using the S97A, K100A, and H363A mutant rLipE proteins. Since Gly^342^ is predicted to be one of the catalytic residues for LipE’s lipase activity, we also examined its impact on LipE’s β-lactamase activity. The S97A and K100A mutations completely demolished rLipE’s β-lactamase activity, whereas the mutation of H363A retained <10% activity (Fig. 4B). The G342A mutation reduced the rLipE β-lactamase activity to <2%. These data suggest that Ser^97^ and Lys^100^ are absolutely required for the β-lactamase activity of rLipE and that Gly^342^ and His^363^ are also important for its β-lactamase activity. We further determined susceptibility of the LipE mutant strain to β-lactam antibiotics using a resazurin microtiter assay (REMA). Compared to the MICs of the tested β-lactam antibiotics against the wild-type and *lipE* complemented strains, the *lipE*::Tn mutation only led to a 2- to 4-fold MIC reduction, suggesting that LipE may not play a major role in resistance to β-lactam antibiotics (Table 1).

**TABLE 1:** MICs of β-lactam antibiotics and rifampin against wild-type, *lipE*::Tn mutant, and *lipE* complemented M. tuberculosis strains

Drug	MIC (μg/ml)		
	Wild-type strain	*lipE*::Tn mutant	*lipE* complemented strain
Ampicillin	240	120	240
Carbenicillin	240	240	240
Cefoxitin	15	7.5	15
Ceftriaxone	30	7.5	30
Cephalothin	30	7.5	30
Rifampin	0.25	0.25	0.25

### Expression of the *lipE* gene is induced under stressed conditions.

**FIG 5:**
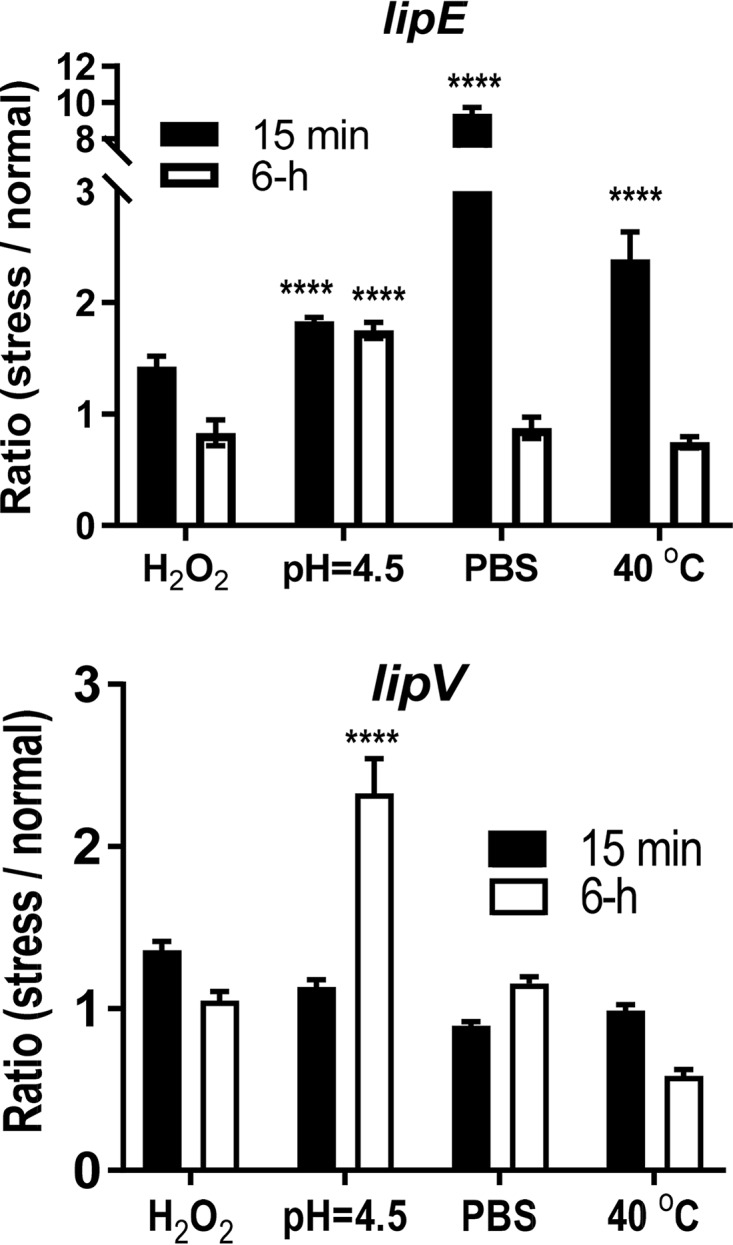
Gene expression of *lipE* under stressed conditions. M. tuberculosis RNA was extracted at 15 min or 6 h after inoculation under the tested conditions. The 16S rRNA gene was used as an internal control to normalize the transcript level of *lipE* under each stressed and normal condition. The gene expression ratio was calculated as the normalized transcript level under the stressed condition/the normalized transcript level under the normal culture condition (MOAD-Tw medium at 37°C). The conditions that induced *lipE* expression significantly are marked with asterisks (****, *P* < 0.0001). *lipV* served as a control, because it was reported to be upregulated only under the acidic stress condition at 6 h. The statistical significance values (*P*) for comparisons between groups are shown in Table S2.

M. tuberculosis inside macrophages is exposed to several stressed conditions, such as reactive nitrogen intermediates and reactive oxygen species. When the phagosomes mature and sequentially fuse with lysosomes, M. tuberculosis faces the highly acidic and hydrolytic milieu of lysosomes (33). We studied *lipE* gene expression under the following four stressed conditions that mimic the M. tuberculosis intracellular niche: (i) oxidative stress (5 mM H_2_O_2_ in regular medium at 37°C); (ii) acidic stress (regular medium at pH 4.5 and 37°C); (iii) nutritive stress (1× phosphate-buffered saline [PBS] buffer at 37°C); and (iv) heat stress (regular medium at 40°C). Bacterial RNA was extracted at 15 min or 6 h after inoculation. The *rv3203* (*lipV*) gene of M. tuberculosis, which encodes another Lip family lipase, was reported to be induced only under acidic stress at 6 h when tested under these four conditions, so it served as a control (34). The 16S rRNA gene was used as an internal control to normalize the expression level under stressed or normal conditions. The gene expression ratio was calculated as the normalized transcript level under the stressed condition divided by the normalized transcript level under the normal culture condition (Fig. 5). Under the nutritive and heat stresses, *lipE* expression was transiently induced [(9.39 ± 0.36)-fold (*P* < 0.0001) and (2.39 ± 0.24)-fold (*P* < 0.0001), respectively], suggesting that LipE was upregulated during the initial adaptation to these two stresses. Under the acidic stress condition, *lipE* expression was induced at both early [(1.84 ± 0.03)-fold, *P* < 0.0001] and late [(1.75 ± 0.07)-fold, *P* < 0.0001] time points. Upregulation of *lipE* expression under these stressed conditions indicates that LipE might have a specific role in surviving one or more of these stressed conditions.

### Mutation of *lipE* leads to attenuated intracellular *M. tuberculosis* growth.

**FIG 6:**
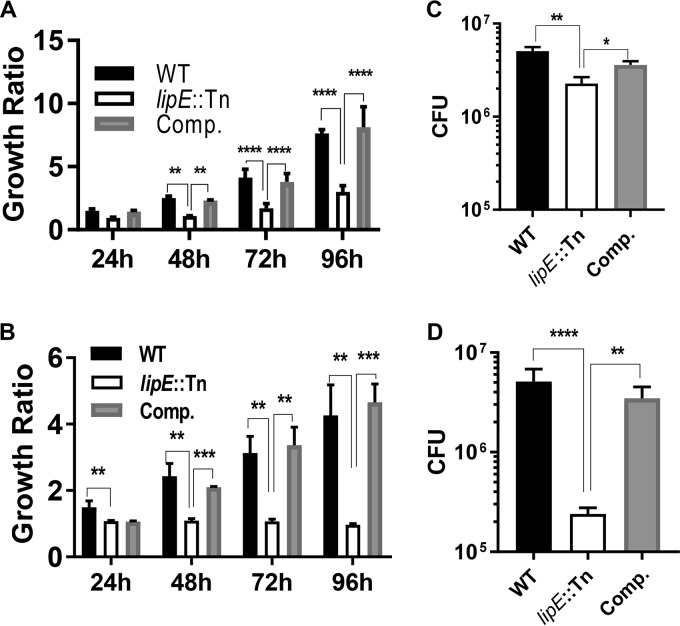
The gene-disrupting mutation of *lipE* leads to attenuated growth in the THP-1 cell line (A and C) and in human PBMC-derived macrophages (B and D). WT, wild type; Comp., complemented (MOI = 10). Intracellular growth ratio = FI at each time point postinfection/FI at 0 h after the extracellular bacteria were removed. Intracellular growth ratios of strains were measured daily for 4 days (A and B), and CFU enumeration was done at 96 h after removal of the extracellular bacteria (C and D). The values represent the means ± the SD of three experiments (****, *P* < 0.0001; ***, *P* < 0.001; **, *P* < 0.01; *, *P* < 0.05 [one-way ANOVA, followed by Dunnett’s multiple-comparison test]).

To determine the role of LipE in M. tuberculosis infection, we acquired a *lipE* transposon insertion mutant strain (point of insertion at bp 320 of the total 1,248-bp *lipE* gene) from BEI Resources. This strain was originally constructed by William Bishai’s laboratory (35). We then constructed the *lipE* complemented strain with this strain. The wild-type, mutant, and complemented strains were labeled with tdTomato fluorescent protein by transforming a tdTomato-expressing plasmid (36). We studied the impact of *lipE*::Tn mutation on M. tuberculosis growth in a common liquid medium, MOAD-Tw, and found that there were no significant differences in growth among the wild-type, mutant, and complemented strains (Fig. S3). We compared cell invasion rates between wild-type, *lipE*::Tn mutant, and complemented strains using the THP-1 cell line. We did not observe significant differences in cell invasion rates among these three strains (Fig. S4). We then studied the impact of *lipE* mutation on M. tuberculosis intracellular growth in infected macrophages. These strains were applied to infect the phorbol myristate acetate (PMA)-stimulated THP-1 cell line and human peripheral blood mononuclear cell (PBMC)-derived macrophages in 96-well plates. The tdTomato-specific fluorescence intensity (FI) was measured at 0 h after removing extracellular bacteria as the baseline fluorescence, and it was then measured daily for 4 days. The intracellular growth ratio of each strain was calculated to normalize the variation in initial numbers of bacteria entering macrophages (Fig. 6A and B). Cells were lysed at day 4 after the fluorescence was determined. The intracellular M. tuberculosis was titrated and plated on 7H11 agar medium for CFU enumeration (Fig. 6C and D). In THP-1 cells, the intracellular growth of the *lipE* mutant strain was significantly lower than that of the wild-type and complemented strains. In human PBMC-derived macrophages, the *lipE* mutant strain also showed significantly less intracellular growth than the wild-type and complemented strains based on both the fluorescence-based growth ratio and the CFU data at day 4.

### Mutation of *lipE* leads to reduced bacterial load in the lungs of infected mice.

**FIG 7:**
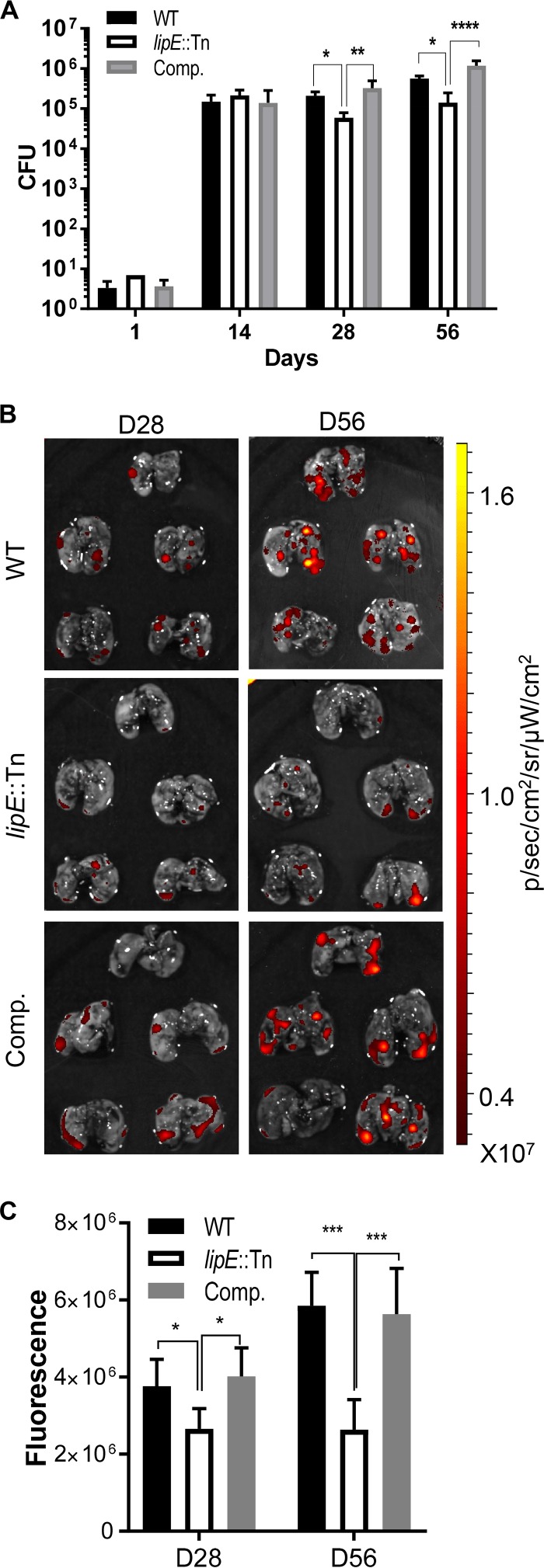
The gene-disrupting mutation of *lipE* leads to a bacterial load reduction in M. tuberculosis-infected mice. (A) CFU of the lungs of mice infected by wild-type, *lipE*::Tn mutant, and complemented M. tuberculosis strains at days 1, 14, 28, and 56. (B) *Ex vivo* imaging of the lungs of mice infected by wild-type, *lipE*::Tn, and complemented M. tuberculosis strains at days 28 and 56 postinfection. (C) Quantitative analysis of *ex vivo* imaging data. Five mice in each group were sacrificed at each time point. WT, wild type; Comp., complemented. ****, *P* < 0.0001; ***, *P* < 0.001; **, *P* < 0.01; *, *P* < 0.05 (one-way ANOVA, followed by Dunnett’s multiple-comparison test).

The cell infection results suggest that LipE is important for M. tuberculosis intracellular survival. We selected C3HeB/FeJ mice as an animal model for M. tuberculosis aerosol infection to determine LipE’s impact on M. tuberculosis *in vivo* infection, because this mouse model develops highly organized granulomas with centered necrotic lesions in the lungs resembling human pulmonary lesions (37). We compared bacterial loads in the lungs of mice at days 14, 28, and 56 postinfection. The mutation of *lipE* led to a significant bacterial load reduction as determined by CFU enumeration compared to the wild-type and complemented strains at days 28 and 56 postinfection (Fig. 7A). Consistent with the CFU data, imaging of the lung tissues from the mice of the three groups using the IVIS Spectrum *in vivo* imaging system (PerkinElmer) showed that the mutant strain-infected mice had significantly lower fluorescence signal from the lungs (Fig. 7B and C). Together, these results suggest that LipE is important for M. tuberculosis intracellular survival and *in vivo* infection.

## DISCUSSION

Our cell and mouse infection data strongly support that LipE is important for M. tuberculosis survival/growth in macrophages and in hosts. In addition, our results demonstrate that LipE is upregulated by a nutrient starvation condition. Similarly, another study also reported that *lipE* was upregulated by a condition that first induced triglycerol accumulation in M. tuberculosis after a 12-day hypoxic growth and then exposed M. tuberculosis to starvation (PBS) (8). Furthermore, the TLC data from our study demonstrate that rLipE can hydrolyze glyceryl trioctanoate, a triglycerol with a medium-carbon chain. Together, these data strongly suggest that LipE might be involved in triglycerol metabolism in M. tuberculosis and contribute to M. tuberculosis intracellular survival. Several M. tuberculosis lipases have been reported to play critical roles in M. tuberculosis virulence. For example, mutation of *lipF* caused bacterial load reduction in the lungs of mice (29). Overexpression of LipY in M. bovis bacillus Calmette-Guérin reduced immune protection against M. tuberculosis in mice (14). Mutation of Rv2224c, another lipase/esterase, also caused a reduction in the bacterial load in mice (10). Recently, we demonstrated that the gene-disrupting mutation of *rv1075c*, which encodes an esterase, significantly attenuated M. tuberculosis intracellular growth in macrophages and reduced the bacterial load in the lungs of aerosol-infected mice (38). Because these lipases/esterases are important for M. tuberculosis infection and growth in hosts, thorough determination of their functions in M. tuberculosis lipid/ester catabolism pathways is necessary to expand our knowledge of TB pathogenesis.

Among the 24 Lip family lipases/esterases of M. tuberculosis, so far only LipE and LipV (34) have been found to preferentially hydrolyze medium-chain triglycerols. Of the three Lip family proteins that were predicted to be evolutionally close to LipE (i.e., LipD, LipL, and LipP), the lipase activities of LipL and LipD have been characterized. LipL prefers a short-chain glyceride butyrate (*k*_cat_ = 0.0932 s^−1^) (15), and LipD preferentially hydrolyzes palmitate (*k*_cat_ = 0.02112 s^−1^) (13). Fatty acids (FAs) released from hydrolysis of medium-chain triglycerols can go through various pathways. For example, FAs released by lipases from medium-chain triglycerols can be synthesized into long-chain FAs by Fas I/II (39). The long-chain FAs are then synthesized into mycolic acids and methyl-branched FAs, which are major components of M. tuberculosis cell membrane and cell envelope (40). The medium-chain FAs can be also converted into precursors through β-oxidation pathways to feed into the tricarboxylic acid (TCA) cycle to provide energy for M. tuberculosis. We speculate that LipE might be involved in hydrolysis of medium-chain triglycerols to generate critical intermediary metabolites to construct M. tuberculosis cell membrane and cell envelope or to feed into the TCA cycle. This hypothesis remains to be further examined.

LipE was identified in the membrane fraction of M. tuberculosis using 2D gel electrophoresis combined with liquid chromatography-mass spectrometry (41). In addition, Malen et al. used 2D gel electrophoresis combined with matrix-assisted laser desorption ionization–time of flight mass spectrometry (MS) and liquid chromatography-coupled tandem MS to profile secreted proteins in M. tuberculosis cell filtrates (42), and LipE was not found among the identified total 257 secreted proteins. We also predicted its subcellular location using several web-based software tools. LipE protein had no predicted signal peptides according to the SignalP 3.0 tool, which indicated that LipE is not secreted via the classical pathway. LipE was predicted to be cell membrane associated or located in the periplasm by Gpos-PLoc (http://www.csbio.sjtu.edu.cn/bioinf/Gpos-multi/), Phobius (http://www.ebi.ac.uk/Tools/pfa/phobius/), and PSORTb 3.0.2 (http://www.psort.org/psortb/index.html). Experimental and *in silico* evidence indicates that LipE is cell membrane associated, and thus it can access both external and internal triglycerols to participate in lipid metabolism.

Protein-BLAST showed that LipE has homologies to class A β-lactamase-related serine hydrolase with unknown function. It is similar to Staphylococcus aureus proteins FmtA and Flp, Burkholderia gladioli esterase EstB, and Bacillus cereus alkaline d-peptidase. We have also identified that it has a domain (aa 43 to 398) of β-lactamase using InterProScan5. LipE has three conserved motifs which are observed in penicillin-binding proteins and β-lactamases, including three SxxK motifs (97SAAK100, 131SHGK134, and 385SSGK388), one SxN motif (282SSN284), and one HLG box (363HLG365). The SxxK motif identified by MyHits (https://myhits.isb-sib.ch/cgi-bin/motif_scan) is conserved in the carboxylesterase VIII family (43). Our site-directed mutagenesis analysis results demonstrate that the β-lactamase activity and lipase activity of LipE share the same catalytic residues. However, the results of the β-lactam antibiotic resistance assay indicate that LipE does not contribute significantly to β-lactam antibiotic resistance of M. tuberculosis. Since LipE’s amino acid sequence shares homology with dd-peptidases, it could have dd-peptidase activity, which remains to be further explored. Our phylogenetic analysis suggests that LipE is evolutionarily related to LipD and LipL. Like LipE, both LipD and LipL have the typical β-lactamase motif SxxK. However, LipD was found to have no β-lactamase activity (13). Although LipL was shown to have both lipase and β-lactamase activities, its *k*_cat_ value for β-lactamase activity was not reported (32). M. tuberculosis has another β-lactamase-encoding gene, *blaC* (*k*_cat_ = 38 ± 2 s^−1^ on nitrocefin), which has been shown to possess a TAT signal sequence and can be exported through the TAT system. BlaC plays a major role in M. tuberculosis resistance to β-lactam antibiotics, since the mutation of *blaC* significantly increases (>16-fold) M. tuberculosis sensitivity to most of the β-lactam antibiotics (44).

We revealed the overall topological organization of LipE through homology modeling. The 3D model of LipE showed that LipE was composed of multiple α-helices and a β-sheet structure, suggesting that LipE belongs to the α/β-hydrolase fold family. We determined that LipE has a 69GHGWG73 motif, which is located in the flexible loop between the second β-sheet and the second α-helix from the N terminus in the predicted 3D structure model. Based on the 3D model, we predicted that the active center of LipE as a lipase is composed of Ser^97^, Gly^342^, and His^363^; this is confirmed by the results of the site-directed mutagenesis experiment. These data provide important information for further detailed analysis of the crystal structure of LipE and its lipase/esterase substrates in M. tuberculosis lipid/ester metabolism.

In summary, we demonstrate here that M. tuberculosis LipE has a lipase/esterase activity and is important for M. tuberculosis intracellular survival and *in vivo* infection. The data from this study imply that LipE is involved in the lipid metabolism of M. tuberculosis during infection. Further thorough studies to precisely determine the substrates of LipE and its roles in M. tuberculosis lipid metabolism and TB pathogenesis will expand our knowledge of how M. tuberculosis appropriates host lipids/esters during infection. Identification of critical enzymes in M. tuberculosis lipid metabolism will unveil M. tuberculosis vulnerabilities to aid drug discovery efforts.

## MATERIALS AND METHODS

### Expression vectors, bacterial strains, and media.

The M. tuberculosis *lipE* transposon-insertion mutant strain was obtained from BEI Resources. All M. smegmatis and M. tuberculosis strains were grown in 7H9 broth (Difco, Detroit, MI) supplemented with 0.5% glycerol, 10% oleic acid dextrose complex without catalase, and 0.05% Tween 80 (MOAD-Tw broth), in Middlebrook 7H9 medium supplemented with 10% oleic acid dextrose complex without catalase and 15 g/liter Bacto agar (MOAD agar; Difco), or on 7H11 selective agar.

### Constructing complemented LipE strain.

The complementation of the *lipE* mutant was achieved using a single-copy, site-specific integrating construct that carries the endogenous promoter for *lipE*. This complementing construct was made by cloning the entire *lipE* locus along with 78 bp upstream of the translational start and 81 bp downstream of the translational end into the HindIII/BamHI sites under the L5 promoter in the integrating vector pYK13, which expresses a tdTomato under the L5 promoter and carries a hygromycin resistance gene. Complementation of *lipE* was confirmed by PCR and DNA sequencing. The wild-type and mutant strains were also transformed with the tdTomato-expressing plasmid pYK13.

### Physiochemical properties.

The amino acid sequence of LipE of M. tuberculosis H37Rv was retrieved from Tuberculist (http://tuberculist.epfl.ch/). Motif determination was carried out using Motif Scan at http://myhits.isb-sib.ch/cgi-bin/motif_scan. Sequence alignment of proteins was carried out by T-COFFEE Espript (http://tcoffee.crg.cat/) with reference to known Lip family lipases of M. tuberculosis, and a conserved region in a defined box was assigned to the aligned file by Espript.3. The evolutionary phylogenetic tree was constructed using the neighbor-joining algorithm in MEGA7.

### Cloning, overexpression, and purification of recombinant LipE. (i) Cloning.

DNAs of Rv3775 (LipE) and pMyC vector were extracted using the Agarose GelExtract minikit (5-PRIME). The primers were designed (listed in Table S1 in the supplemental material) to amplify *lipE* from the genomic DNA of M. tuberculosis CDC1551 according to the following program: denaturation for 5 min at 95°C; followed by 34 cycles of 30 s at 95°C, 30 s at annealing temperature (70°C), and 1 min 30 s at 72°C; and then 5 min at 72°C for the ﬁnal extension. Further PCR-ampliﬁed product was analyzed on a 0.8% agarose gel and puriﬁed by using a DNA Clean & Concentrator kit (Zymo Research). Eluted puriﬁed PCR products and DNA of the expression vector pMyC (Addgene) were digested with BamHI and HindIII, respectively. The restriction enzyme-digested pMyC vector fragments were dephosphorylated by phosphatase. After electrophoresis, the DNA bands were cut off the gel and purified, and the gel-purified DNA fragments (vector and ampliﬁed *lipE* PCR product) were mixed and ligated with the T4 DNA ligase. The ligated product was transformed to the chemically competent Escherichia coli DH5α cells for amplification. The cloned *lipE* was further conﬁrmed by sequencing (Eurofins Genomics). For the expression of LipE, the constructed plasmid was isolated and transformed into the expression host M. smegmatis mc^2^155 cells. Positive transformants were screened on MOAD agar plates containing 80 μg/ml hygromycin and confirmed by DNA sequencing.

### (ii) Overexpression of LipE.

The transformant of M. smegmatis was cultured in MOAD-Tw broth until an optical density at 600 nm (OD_600_) of 1.5 was reached, induced for 16 h by the addition of 0.2% acetamide, and harvested by centrifugation at 3,000 × *g* for 15 min. The pellet was resuspended in cold lysis buffer containing 20 mM Tris-HCl (pH 7.5), 100 mM NaCl, 5% glycerol, 1 mM imidazole, 10 mM β-mercaptoethanol, and 1× protease inhibitor cocktail (Sigma-Aldrich) and then broken with a French press. The His-tagged rLipE protein was purified by Ni-nitrilotriacetic acid (Ni-NTA) agarose column-based affinity chromatography and eluted with 10 mM Tris buffer (pH 8.0) containing 300 mM NaCl and a concentration gradient of imidazole. Eluted fractions containing purified rLipE were analyzed by SDS-PAGE.

### Lipase activity assay.

The enzymatic assay of purified recombinant rLipE was performed using different *p*-nitrophenyl (*p*-NP) ester substrates such as acetate (*p*-NP-C2), butyrate (*p*-NP-C4), hexanoate (*p*-NP-C6), caprylate (*p*-NP-C8), caprate (*p*-NP-C10), laurate (*p*-NP-C12), myristate (*p*-NP-C14), palmitate (*p*-NP-C16), and stearate (*p*-NP-C18). The 50 mM stock solutions of substrates were prepared in absolute ethanol. The enzymatic reaction mixture was total 350 μl and contained 2 mM *p*-NP ester substrate, 50 mM phosphate buffer (pH 7.4), 2 mM sodium-deoxycholate, and 10 μg of purified rLipE. The reaction mixture was loaded into a 96-well plate in triplicates with 100 μl/well, and the negative control was the same mixture, except that the rLipE was replaced with the buffer. The enzymatic hydrolysis product of all the substrates was *p*-NP that was quantified by measuring the absorbance at 405 nm kinetically using a spectrophotometer (BioTek Synergy 2) at various programed temperatures with a path-length correction to 1 cm using a constant of 0.18. During data analysis, the absorbance of the negative control was subtracted from the absorbance of samples with rLipE. One unit of enzyme activity is defined as the amount of enzyme that generates 1 μmol of *p*-NP from a *p*-NP ester substrate per min under the standard assay condition. Enzyme activity of the purified rLipE was determined as a function of the substrate concentration (0.01 to 1.0 mM *p*-NP-C6) using the standard assay method at 37°C. The Michaelis-Menten constant (*K_m_*) and the maximum velocity for the reaction (*V*_max_) were calculated.

### Effects of pH and temperature on enzyme activity.

To find out pH optima for rLipE’s esterase activity, an enzyme assay was performed in buffers (50 mM) with different pH values ranging from pH 4.0 to 11.0. Acetate buffer (pH 4.0 to 5.0), phosphate buffer (pH 6.0 to 8.0), Tris-HCl buffer (pH 8.0 to 9.0), and glycine-NaOH buffer (pH 10.0 to 11.0) were used. To determine optimal temperature of the enzyme activity, the enzyme assay was performed at various temperatures (25 to 50°C) for 15 min. The enzyme activity was determined as described above.

### Site-directed mutagenesis.

Site-directed mutagenesis was carried out by substituting conserved putative active-site residues Ser^97^, Lys^100^, Gly^342^, and His^247^ with alanine using a site-directed mutagenesis kit (QuikChange II; Agilent, Santa Clara, CA). The primers are listed in Table S1 in the supplemental material. XL-1-Blue Supercompetent E. coli cells were transformed with plasmids (pMyc backbone) having a confirmed mutant *lipE* gene. The resulting plasmids were transformed into M. smegmatis, and the mutant proteins were overexpressed as described above. The mutant rLipEs were purified, checked for enzymatic activity, and compared to the wild-type enzyme.

### Thin-layer chromatography.

Trioctanoate (Sigma-Aldrich) at 50 mM was incubated with 100 μl of the wild-type or mutant rLipE (2.5 mg/ml) in a 500-μl reaction mixture for 30 min. The negative controls did not have rLipE added but had an equal volume of buffer added instead. Lipids were extracted with a 2:1 mixture of chloroform-methanol, followed by gentle mixing and centrifugation at 10,000 rpm for 5 min. The extracted products were run on silica-coated TLC plates. Trioctanoate without rLipE and the monooctanoate (Sigma-Aldrich) or dioctanoate (Avanti) served as standards. Chloroform-acetic acid (95:5) was applied as a mobile phase and Primuline stain (Sigma-Aldrich; 5 mg/100 ml in acetone-water [80:20]) under 384-nm UV light was used to visualize the products on TLC.

### REMA for MIC.

Ampicillin, carbenicillin, cefoxitin, ceftriaxone, cephalothin, and rifampin were purchased from Sigma-Aldrich. The M. tuberculosis inoculum was prepared from log-phase M. tuberculosis strains (i.e., wild-type CDC1551, *lipE*::Tn mutant, and *lipE* complemented strains) growing in MOAD-Tw medium. After we adjusted the absorbance of the bacterial culture to a McFarland tube no. 1, the bacteria were diluted 1:20 with the medium, and 100 μl was used as an inoculum. After being loaded with various concentrations of each antibiotic, the plates were covered, sealed in plastic bags, and incubated at 37°C in a normal atmosphere. After 7 days of incubation, 30 μl of resazurin solution (0.02%) was added to each well, followed by incubation overnight at 37°C, and the samples were then assessed for color development. A change from blue to pink indicated the reduction of resazurin and therefore bacterial growth. The MIC was defined as the lowest drug concentration that prevented this color change. All MICs were performed in duplicate on at least two independent cultures.

### Prediction of a 3D model and refinement of the model.

The templates for homology modeling were selected based on sequence identity and query coverage in SWISS-MODEL workspace. The top three 3D model structures were further screened through PyMOL software to select the lowest RMSD value and hydrogen bond distance between the catalytic residues on structural alignment with the template and further energy minimization by YASARA force ﬁeld.

### *lipE* gene expression under several stress conditions.

The M. tuberculosis CDC1551 strain was grown to mid-log phase and harvested by centrifugation. The bacterial pellet was resuspended in different media for exposure to stressed conditions such as oxidative stress (MOAD-Tw broth with 5 mM H_2_O_2_), acidic stress (MOAD-Tw broth at pH 4.5), nutrient stress (1× PBS), and temperature stress (40°C). These stressed cultures were grown for 15 min or 6 h along with a control culture in normal MOAD-Tw broth. After centrifugation, the pellets were resuspended in 1 ml of TRIzol to isolate RNA using a Direct-zol RNA miniprep kit (Zymo Research) according to the manufacturer’s instructions. The integrity, quantity, and quality of RNA samples were validated by using an Agilent bioanalyzer.

### Reverse transcription.

A RevertAid RT kit (Thermo Scientific) was used for reverse transcription of RNA to cDNA. The random hexamer primer (1 μl) and template RNA (1 μg) were first incubated at 65°C for 5 min and then chilled on ice. After being spun down, 5× reaction buffer (4 μl), RiboLock RNase inhibitor (1 μl), 10 mM deoxynucleoside triphosphate mix (2 μl), and RevertAid RT (200 U/μl, 1 μl) were added to the reaction mixture in a total 20-μl volume. The reaction mixture was incubated for 5 min at 25°C, followed by 60 min at 42°C. The reaction was then terminated by heating at 70°C for 5 min. The cDNA samples were diluted to 1:10, 1:20, and 1:100 to further determine the optimal dilution in the quantitative PCR (qPCR) analysis.

### qPCR.

The 16S rRNA gene was used as an internal control. The reaction mixture consisted of cDNA (2 μl), primers for *lipE*, 16S rRNA gene, or *lipV* F and R (see Table S1 in the supplemental material) at 10 μM (0.2 μl each), 2× KAPA SYBR FAST qPCR Master mix (5 μl), and water (2.6 μl), for a total of 10 μl. The reaction mix was loaded into a 96-well qPCR plate and amplified by a LightCycler 480 (Roche) at the Molecular Resource Center of the University of Tennessee Health Science Center (UTHSC) using the following program: denaturation for 5 min at 95°C, followed by 40 cycles of 10 s at 95°C, 30 s at 60°C, and 10 s at 72°C.

### Data analysis.

For each M. tuberculosis culture condition, we utilized three biological replicates. For each cDNA sample, we placed four technical replicates on the sample 96-well plate. For each condition, the Δ*C_T_* is equal to the threshold cycle (*C_T_*) value of *lipE* (or *lipV*) after subtraction of the *C_T_* value of the 16S rRNA gene under each condition. The ΔΔ*C_T_* was calculated as the Δ*C_T_* of each stressed condition after subtraction of the Δ*C_T_* for the normal culture condition. The expression fold change was calculated as the 2^–ΔΔ^*^CT^* value.

### Cell infection.

The tdTomato expressing the M. tuberculosis CDC1551 wild-type, *lipE*::Tn mutant, and *lipE* complemented strains was cultured in MOAD-Tw medium until it reached the log phase and applied to infect macrophages. THP-1 cells or human PBMC-derived macrophages were seeded into 96-well transparent-bottom culture plates at a concentration of 5 × 10^4^ cells/well. For THP-1, PMA was used to stimulate the cells for 3 days. Human PBMCs acquired from the American Type Culture Collection were seeded into 96-well plates for 7 days to allow monocytes to develop into macrophages and to remove the suspending T-cells. The THP-1 or human PBMC-derived macrophages were then infected by bacteria at a multiplicity of infection (MOI) of 10, with three replicates for each strain, at 37°C for 3 h. The extracellular bacteria were aspirated, and the infected cells were washed with 1× PBS and then treated with amikacin (200 μg/ml) for an additional 2 h. After being washed with 1× PBS again, the plate was loaded into a microplate reader (Tecan Infinite 200 Pro) to measure the fluorescence with tdTomato-specific excitation (550 nm) and emission (590 nm) wavelengths. At day 4 postinfection, the cell culture medium was removed and replaced with 100 μl of 0.1% Triton X-100 in H_2_O for 10 min to lyse the cells. Bacteria from lysed cells were titrated and plated on 7H11 agar plates. After 3 to 4 weeks of culture at 37°C, the colonies on the agar plates were counted.

### Mouse infection.

C3HeB/FeJ female mice (Jackson Laboratory, Bar Harbor, ME), aged 6 to 8 weeks, were aerosol infected using a bioaerosol nebulizing generator (BANG; CH Technologies, Inc.) at the regional biocontainment laboratory of the UTHSC with the M. tuberculosis wild-type, *lipE*::Tn mutant, and *lipE* complemented strains from log-phase cultures to deliver a low dose (5 to 10 CFU) of M. tuberculosis into the lungs at day 0. At day 1, two mice from each group were sacrificed to determine the actual delivered bacterial number in the lungs. At days 14, 28, and 56, five mice per group were sacrificed for tissue collection. Because all three M. tuberculosis strains expressed tdTomato, we conducted *ex vivo* fluorescence imaging with the harvested lungs using IVIS imaging. The tissues were then weighed and homogenized to determine the CFU using 7H11 selective agar plates.

### Ethics statement.

The UTHSC Institutional Animal Care and Use Committee (IACUC) approved animal care and use protocol 16-102 for all animal experiments in this study. The UTHSC IACUC adheres to the Public Health Service Policy and Animal Welfare Act.

## Supplementary Material

Supplemental file 1
