# Supplementary material for: Mycobacterium tuberculosis LipE Has a Lipase/Esterase Activity and Is Important for Intracellular Growth and In Vivo Infection
Source: Infect Immun. 2019 Dec 17;88(1):e00750-19. doi: 10.1128/IAI.00750-19 (PMC6921666; doi:10.1128/IAI.00750-19)
Supplement: Supplemental file 1 [file IAI.00750-19-s0001.pdf]

SUPPLEMENTAL DATA:

Table S1: Primers used in this project.

Primers for expressing and purifying protein	
Rv3775P-pMyc-F: 5' GA GGATCC ATGCGCGCTGGCGAC 3'	Rv3775P-pMyc-R: 5' GA AAGCTT GGCCCGACGATGCCCGTGG 3'
Primers for constructing complementary strain	
RV3775 F: 5' TATA AAGCTT AACGTT CTTAAG CAAGGACCTCACCGAAGGTA 3'	RV3775 R: 5' TATA GGATCC TTAATTAA CATATG ATAAGGCAACAGGCCGACAT 3'
Primers for site directed mutagenesis	
S97A-F: 5' GTTCTGCGTGTACGCGGCGGCCAAGGC 3'<br>K100A-F: 5' GTACTCGGCGGCCGCGGCGATCACGGCG 3'<br>G342A-F: 5' GCTGGGGCACCGCGTTCATGCTGGG 3'<br>H363A-F: 5' GGCGGCATTCGGCGCTCTCGGTCTGGTC 3'	S97A-R: 5' GCCTTGGCCGCCGCGTACACGCAGAAC 3'<br>K100A-R: 5' CGCCGTGATCGCCGCGGCCGCCGAGTAC 3'<br>G342A-R: 5' CCCAGCATGAACGCGGTGCCCCAGC 3'<br>H363A-R: 5' GACCAGACCGAGAGCGCCGAATGCCGCC 3'
Primers used in stress-induced expression study	
qPCR-LipE-F: 5' ATGAATACGCGGTGGAAAGG 3'<br>qPCR-16S rRNA rrs-F: 5' CCGGAATTACTGGGCGTAAA 3'<br>LipV-Rv3203-F: 5' GAGCACCTCGTCGCATT 3'	qPCR-LipE-R: 5'GTAGACGATCTCACGCATCAAC 3'<br>qPCR-16S rRNA rrs-R: 5' AGTACTCTAGTCTGCCCGTATC 3'<br>LipV-Rv3203-R: 5' GCTCCAGTAGCACACCATC 3'

Table S2: Statistical significance (*P* values) for comparisons of *lipE* and *lipV* expression levels between different conditions at the same time point, and between time points under the same condition.

**LipE**	**15-min**	**6-h**	**15-min vs. 6-h**
H_2O_2 vs. pH=4.5	0.0008	<0.0001	
H_2O_2 vs. PBS	<0.0001	0.5709	
H_2O_2 vs. 40 °C	0.0003	0.2279	
pH=4.5 vs. PBS	<0.0001	<0.0001	
pH=4.5 vs. 40 °C	0.0118	<0.0001	
PBS vs. 40 °C	<0.0001	0.0546	
H_2O_2			0.0002
pH=4.5			0.1200
PBS			<0.0001
40 °C			<0.0001
**LipV**	**15-min**	**6-h**	**15-min vs. 6-h**
H_2O_2 vs. pH=4.5	<0.0001	<0.0001	
H_2O_2 vs. PBS	<0.0001	0.0249	
H_2O_2 vs. 40 °C	<0.0001	<0.0001	
pH=4.5 vs. PBS	0.0003	<0.0001	
pH=4.5 vs. 40 °C	0.0052	<0.0001	
PBS vs. 40 °C	0.0053	<0.0001	
H_2O_2			0.0002
pH=4.5			0.0003
PBS			<0.0001
40 °C			<0.0001

Note: *P* values were determined from non-paired *t*-tests.

Fig. S1.

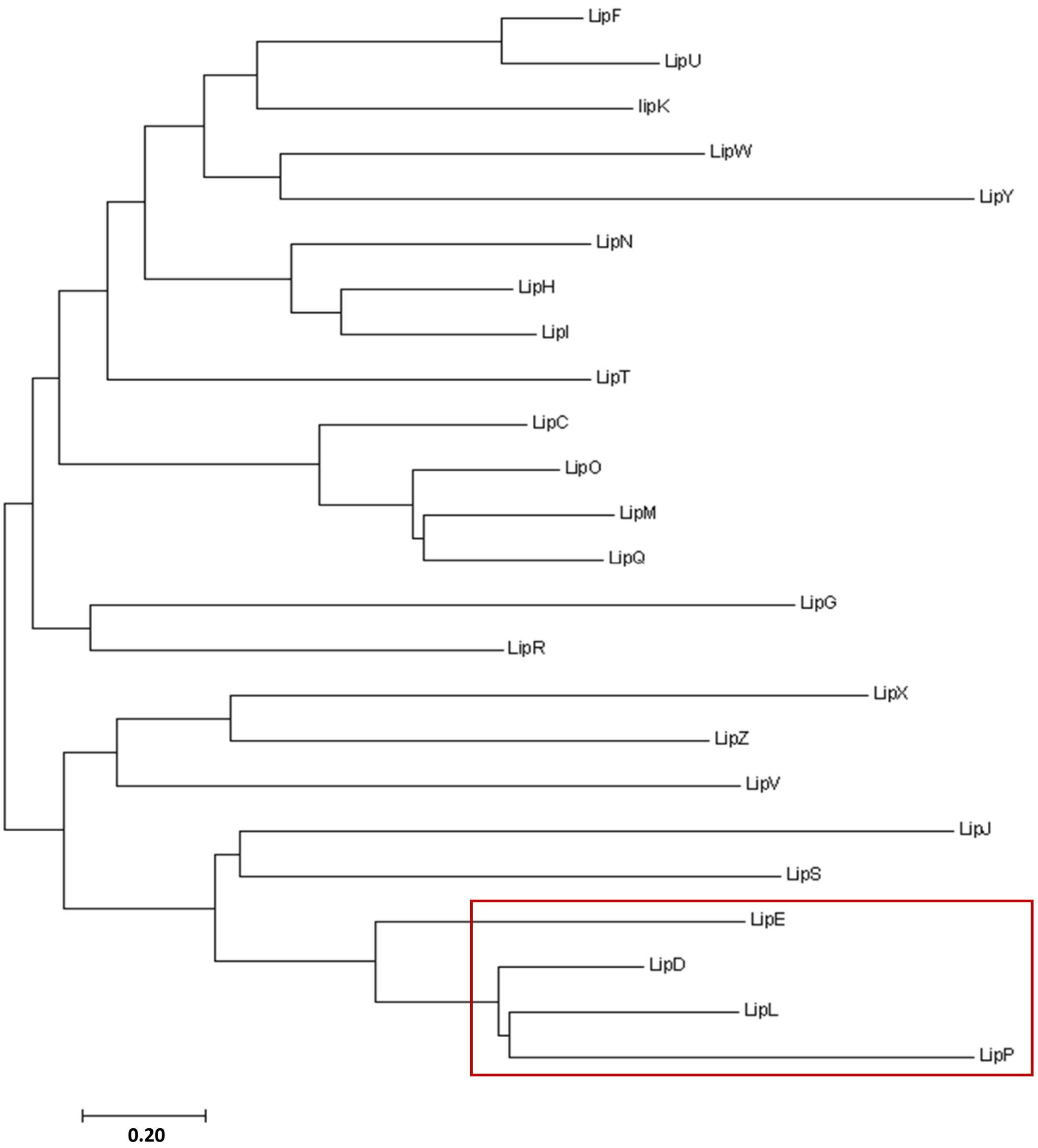

Fig. S1. Phylogenetic relationships of *Mtb* LipE to other Lip family proteins. The analysis was performed with MEGA7.0 using Neighbor-Joining algorithm. The red box highlights the subgroup of LipE, LipD, LipL and LipP.

Fig. S2.

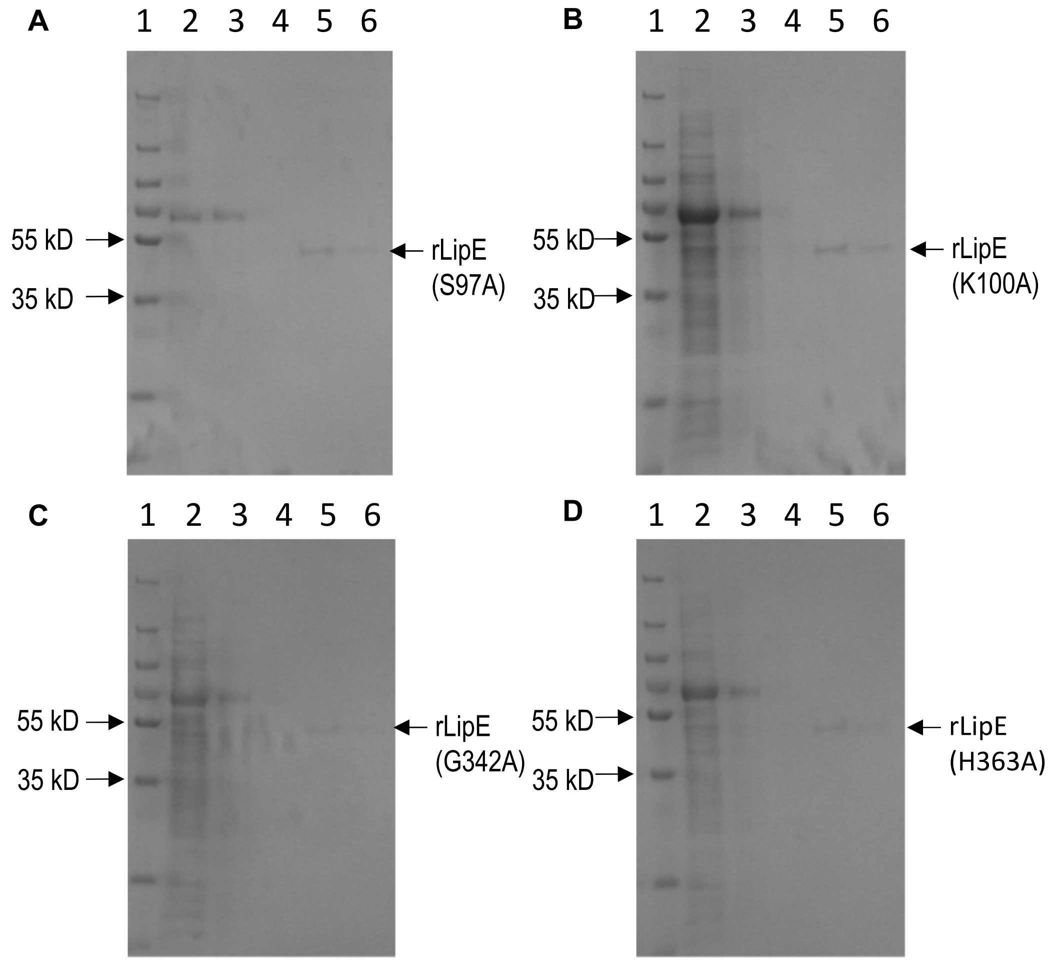

Fig. S2. SDS-PAGE analysis mutant rLipEs eluted by various concentrations of imidazole from His-tag Ni-column. A. rLipE S97A; **B.** rLipE K100A; **C.** rLipE G342A; and **D.** rLipE H363A. Lane 1: Marker (PageRuler prestained); Lane 2: protein extracts run through the Ni-column in 40 mM imidazole buffer; lane 3-6, samples eluted from Ni-column with 62.5, 125, 250, and 500 mM imidazole, respectively.

Fig. S3.

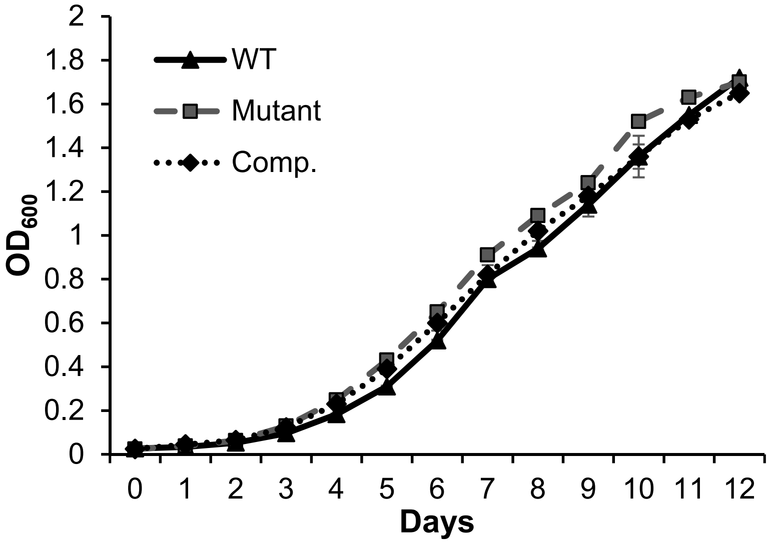

Fig. S3. Comparison of growth between the wild-type, *lipE*::Tn mutant, and complemented strains in a common liquid medium. The CDC1551 wild-type, *lipE*::Tn mutant, and *lipE* complemented strains were inoculated into the MOAD-Tw liquid medium and cultured at 37 °C for 12 days.

Fig. S4.

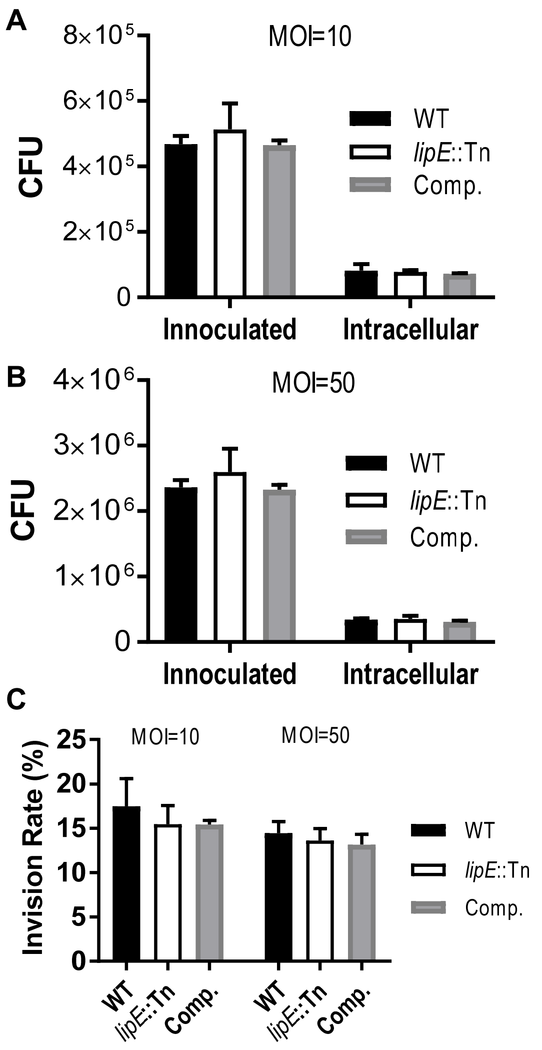

Fig. S4. Comparison cell invasion rates between the wild-type, *lipE*::Tn mutant, and complemented strains. THP-1 derived macrophage was infected with the three strains at MOI 10 (**A**) or 50 (**B**). The invasion rates of the three strains were compared (**C**). CFU enumeration was conducted at beginning of the infection and 3-h post infection after removing extracellular Mtb.
